# Regulator of oligodendrocyte maturation, miR-219, a potential biomarker for MS

**DOI:** 10.1186/s12974-017-1006-3

**Published:** 2017-12-04

**Authors:** Ilona B. Bruinsma, Marie van Dijk, Claire Bridel, Timothy van de Lisdonk, Sanne Q. Haverkort, Tessel F. Runia, Lawrence Steinman, Rogier Q. Hintzen, Joep Killestein, Marcel M. Verbeek, Charlotte E. Teunissen, Brigit A. de Jong

**Affiliations:** 1 Department of Neurology, Donders Institute for Brain Cognition and Behaviour, Radboud University Medical Center, Nijmegen, the Netherlands; 2 Department of Laboratory Medicine, Radboud University Medical Center, Nijmegen, the Netherlands; 3 Molecular Biology Laboratory, Department of Clinical Chemistry, VU University Medical Center, Amsterdam, the Netherlands; 4 Neurochemistry Laboratory and Biobank, Department of Clinical Chemistry, Amsterdam Neuroscience, VU University Medical Center, Amsterdam, the Netherlands; 5 Department of Neurology and Department of Immunology, Erasmus MC, Rotterdam, the Netherlands; 6 Department of Neurological Sciences and Neurology, Stanford University, Stanford, CA USA; 7 Department of Neurology, Amsterdam Neuroscience, VUmc MS Center Amsterdam, VU University Medical Center, Amsterdam, the Netherlands

**Keywords:** Multiple sclerosis, Cerebrospinal fluid, Biomarkers, miRNA, miR-219, miR-150

## Abstract

**Background:**

Multiple sclerosis (MS) is a demyelinating and degenerative disease of the central nervous system. Normally, demyelination is followed by remyelination, which requires repopulation of a demyelinated area by oligodendrocyte precursor cells. Although large numbers of precursor cells are present in MS lesions, remyelination often fails, in part by the inability of precursor cells to differentiate into mature myelin-forming cells. In mouse and rat, miR-219 is required for this differentiation. Previously, we identified decreased miR-219 expression in tissue of MS patients compared to controls. Cell-free miRNAs have been detected in many different body fluids including cerebrospinal fluid (CSF) and may reflect disease processes going on in the central nervous system. This prompted us to investigate the biomarker performance of CSF miR-219 for MS diagnosis.

**Methods:**

Quantitative PCR was performed measuring miR-219 levels in CSF of MS patients and controls in three independent cohorts.

**Results:**

All three cohorts of MS patients and controls revealed that absence of miR-219 detection in CSF is consistently associated with MS.

**Conclusions:**

We have been able to identify and validate absence of miR-219 detection in CSF of MS patients compared to controls, suggesting that it may emerge as a candidate biomarker for MS diagnosis.

## Background

Multiple sclerosis (MS) is the most common chronic inflammatory demyelinating central nervous system (CNS) disease of young adults worldwide [1]. Although MS has classically been considered a white matter disease, gray matter demyelination is now recognized as an important feature of MS, particularly during the progressive stage of the disease, when neurodegenerative mechanisms predominate [2, 3]. Focal demyelinated plaques throughout the CNS are the major pathological hallmark of MS [4]. Under physiological conditions, demyelination is followed by remyelination, which requires repopulation of a demyelinated area by oligodendrocyte precursor cells (OPCs) [5]. Although large numbers of OPCs are present in MS lesions, remyelination fails, possibly caused by the inability of OPCs to differentiate into mature myelin-forming cells [6, 7]. Lack of remyelination is one major mechanism underlying neurodegeneration in MS [8].

Modulation of gene expression by microRNAs (miRNAs) has a central role in neurodegenerative processes and immune responses [9]. miRNAs regulate gene expression by binding target mRNAs and inhibiting protein synthesis or causing mRNA degradation [9]. There is evidence for dysregulated miRNA expression levels in blood, cerebrospinal fluid (CSF), and white matter tissue of MS patients [10–12]. Biomarkers assisting MS diagnosis and subtyping into relapsing remitting (RR), secondary progressive (SP), and primary progressive (PP) are needed [13]. In this respect, CSF biomarkers based on miRNA expression levels are interesting as they may reflect disease processes going on in the CNS.

In a previous study, we identified a large number of differentially expressed miRNAs in chronic white matter and gray matter lesions of MS patients compared to age-matched healthy controls (Bruinsma et al., submitted). In particular, decreased miR-219 expression was identified in both white matter and gray matter lesions. In mouse and rat, miR-219 is required for both oligodendrocyte differentiation and myelination [14–18]. Downregulation of miR-219 in MS may thus contribute to impaired OPC differentiation and consecutive lack of remyelination in MS. In this study, we therefore assessed the biomarker performance of CSF miR-219 for MS diagnosis.

## Methods

### Human CSF samples

Lumbar CSF samples were obtained from the Radboud UMC, Nijmegen, the Netherlands (cohort 1), the Erasmus University Medical Center, Rotterdam, the Netherlands (cohort 2), and the VU University Medical Center, Amsterdam, the Netherlands (cohort 3). CSF samples were collected, prepared, and stored in agreement with international consensus guidelines [19]. Table 1 shows the demographic and clinical characteristics of the included participants in all three cohorts. All CSF donors signed written informed consent for use of material and clinical information for research purposes.

**Table 1:** Demographic and clinical characteristics of multiple sclerosis patients and controls included in the CSF experiments

Cohort	*N*	Gender (male/female)	Mean age ± SD in years	Mean disease duration ± SD in years	
1	24				
RRMS	7	2/5	40.4 ± 8.9	7.4 ± 5.4	
SPMS	5	1/4	47.8 ± 5.6	21.6 ± 10.5	
PPMS	4	3/1	47.0 ± 9.2	6.4 ± 2.6	
NHI	8	3/5	40.0 ± 6.1	NA	
2	115				
CIS	12	3/9	37.9 ± 9.4	ND	
RRMS	15	3/12	41.6 ± 5.0	6.0 ± 5.7	
SPMS	12	4/8	49.1 ± 9.2	15.3 ± 9.9	
PPMS	11	4/7	49.9 ± 11.9	6.5 ± 4.4	
NHI	34	12/22	53.8 ± 14.8	NA	
IND	4	0/4	50.2 ± 16.8	ND	
AD	17	8/9	70.4 ± 9.1	ND	
IIH	10	1/9	31.4 ± 6.7	ND	
Cohort	N	Gender (male/female)	Median age (interquartile range) in years	EDSS	Interferon-beta used (%)
3	112				
RRMS	44	18/26	40.6 (34.6–49.9)	3.0 (2.5–4.0)	36.4
SPMS	33	19/14	48.1 (43.9–53.8)	6.0 (4.0–6.8)	36.4
PPMS	13	8/5	52.2 (47.6–56.3)	4.0 (3.8–6.3)	0
IND	6	4/2	37.8 (23.1–60.1)	2.5^a^	0
NIND	16	10/6	43.2 (31.3–54.1)	1.0^a^	0

*CIS* clinically isolated syndrome, *RRMS* relapsing remitting MS, *SPMS* secondary progressive MS, *PPMS* primary progressive MS, *NHI* neurologically healthy individual, *AD* Alzheimer’s disease, *IIH* idiopathic intracranial hypertension, *NIND* non-inflammatory neurological disease, *IND* inflammatory neurological disease, *EDSS* Extended Disability Status Scale, *NA* not applicable, *ND* not determined

^a^EDSS measured in one patient only

MS and CIS were diagnosed according to state-of-the-art diagnostic criteria at time of CSF collection [20, 21], and further classified as having relapsing remitting (RRMS), secondary progressive (SPMS), or primary progressive (PPMS) according to Lublin and Reingold [22]. Control groups consisted of individuals with other non-inflammatory neurological diseases (NIND), inflammatory neurological diseases other than MS (IND), Alzheimer’s disease (AD), and idiopathic intracranial hypertension (IIH). Neurologically healthy individuals (NHI) had been assessed for a neurological disorder but were diagnosed with either a systemic disease without neurological manifestations, or, for example, (tension-type) headache.

### RNA isolation from CSF samples

CSF samples (0.5 ml) of cohorts 1 and 2 were spiked with 1 μg MS2 carrier RNA (Roche Applied Science) to increase the yield of RNA isolation [23]. Liquid was removed by freeze-drying on an Alpha 1–2 LDplys freeze dryer (Christ, Osterode am Harz, Germany) and samples were resuspended with 200 μl RNase-free water. RNA was isolated using the miRCURY RNA Isolation kit for biofluids (Exiqon, Vedbaek, Denmark). Equivalent CSF volumes were used as input for the RNA isolation. The expression profiles of miR-24 and miR-16 have been reported to be stable in several bodily fluids and tissues [24–27], and were therefore used for normalization purposes.

RNA isolation of cohort 3 samples was performed on 300 μl CSF again using the miRCURY RNA isolation kit for biofluids (Exiqon, Vedbaek, Denmark) according to the manufacturer’s protocol and including the on-column DNase treatment. To optimize RNA yield per sample, 2 μg glycogen carrier was added to the lysis solution. Secondly, to monitor RNA isolation and proper reference miRNA normalization, per sample, 150 pmol synthetic UniSP6 RNA spike-in (Exiqon) was added to the lysis solution. Eluted RNA was directly stored at − 80 °C.

### Reverse transcription and pre-amplification in CSF samples

The RT reaction in RNA samples of cohorts 1 and 2 was performed as previously described [23] using the Taqman MicroRNA RT Kit (Life Technologies, Landsmeer, the Netherlands) and individual miRNA RT primers in a specific stem-loop conformation. Individual reverse transcription (RT) and quantitative polymerase chain reaction (qPCR) primers for miRNAs (hsa-miR-16, hsa-miR-24, hsa-miR-219) were obtained from Life Technologies. RT products were directly subjected to a pre-amplifaction step using Taqman PreAmp Master Mix (Life Technologies) and individual Taqman miRNA primers for qPCR. The procedure was carried out according to the manufacturer’s protocol for multiplexed pre-amplification. The pre-amplification product was then diluted eight times in RNAse-free 0.1× TE (1 mM Tris-HCl pH 8.0/100 μM EDTA) buffer. First strand cDNA synthesis on the RNA samples of cohort 3 was performed using the Universal cDNA synthesis kit II (Exiqon) according to the manufacturer’s protocol. Per sample 1 μl RNA was used in a 10 μl reaction mixture. An inter-plate control (IPC) was created by pooling RNA of 10 samples and used for cDNA synthesis. To control for potential DNA contamination the IPC reactions were also performed omitting the enzyme mix (−RT). cDNA samples were aliquoted and stored at − 20 °C until PCR.

### Quantitative PCR in CSF samples

For samples of cohorts 1 and 2, 5 μl of diluted pre-amplification products of CSF was used according to the manufacturer’s procedure with Taqman Universal Master Mix II (Life Technologies) and individual Taqman miRNA primers for qPCR. Samples were measured in duplicate. Amplification was performed using a qPCR Thermal Cycler (Applied Biosystems, Nieuwerkerk aan den IJssel, The Netherlands) with a denaturation step at 95 °C for 10 min, followed by 50 cycles of 95 °C for 15 s and 60 °C for 60 s. Data of these qPCR experiments were normalized using the geometric mean [26, 28] of the two uniformly expressed miRNAs, i.e., miR-16 and miR-24. Relative expression levels (REL) were calculated using the formula REL = 2 ^− ∆Ct^, where Ct is cycle threshold, and ∆Ct = Ct (miRNA) – *C*t (geometric mean of miR-16 and miR-24).

Quantitative PCR (qPCR) on the cDNA samples of cohort 3 was performed using the ExiLENT SYBR Green master mix (Exiqon) in combination with primer-mixes for UniSP6, miR-150, miR-219, and miR-204 (Exiqon). cDNA samples were diluted 10× of which 1 μl was added to each 10-μl reaction. qPCRs were done according to the manufacturer’s protocol with the addition of 0.2 μl ROX reference dye (Invitrogen, Waltham, MA, USA) per reaction. All samples were run in triplicate on an ABI7300. On each 96-well plate, the IPC, the IPC-RT, and non-template controls were included in triplicate. Raw Ct (cycle threshold) values were extracted; mean and SDs were calculated for each sample and adjusted using the mean IPC Ct values. Sample values were only included for further analysis when at least two measurements were detectable with less than two Ct difference. Based on the study performed by Bergman and coworkers [29], miR-204 Ct values were used for normalization after confirming high correlation with UniSP6 spike-in Ct levels (Spearman’s rho *p* < 0.0001, *r* = 0.7093), qualifying miR-204 as a reference miRNA in CSF. Relative miRNA expression levels were calculated using REL = 2^− ∆Ct^, where ∆Ct = Ct target miRNA – Ct miR-204. To identify the causes for samples to be undetectable by qPCR for the miRNAs under investigation, the levels of UniSP6 and miR-204 were used. The minimally required RNA isolation yield was determined by identifying the highest detectable Ct value of UniSP6 providing detectable Ct values for each miRNA separately. The minimally required total miRNA level was determined by identifying the highest detectable Ct value of miR-204 providing detectable Ct values.

### Statistical analysis

Statistical differences were determined using the ANOVA procedure in SPSS 20.0 for Windows Software (SPSS Inc., Chicago, IL, USA). Using cross tabulation, the incidence of MS and/or CIS patients with undetectable miR-219 expression was compared with that in control patients by calculating odds ratios (OR) with 95% confidence intervals (CIs) and its *p* value (two-tailed Fisher Exact test). OR above 1 denotes a positive association between the non-measurability of miR-219 expression and MS. As data were not normally distributed as tested with the Shapiro-Wilk test, differences between multiple groups or two groups were analyzed using the Kruskall-Wallis test and the Mann-Whitney *U* test, respectively. Correlations were tested using Spearman’s non-parametric correlation coefficient. For estimation of a biasing effect of age, analysis of variance (ANOVA) on the ranks was applied with age as covariate. A test was considered significant when *p* < 0.05.

## Results

### Absence of CSF miR-219 expression correlates with MS diagnosis in three independent cohorts

**Fig. 1:**
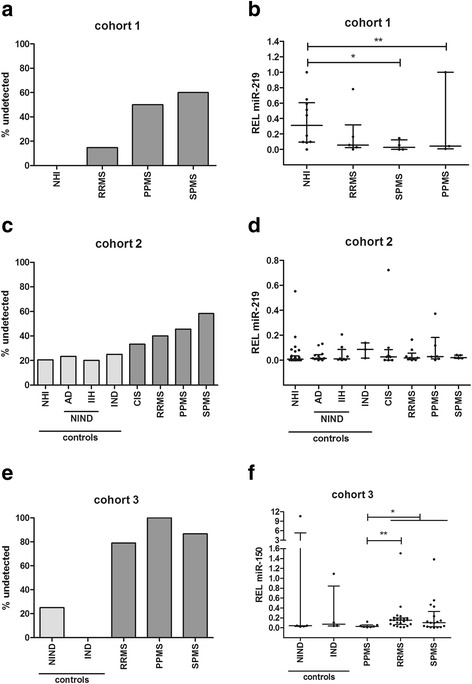
Expression of miR-219 in CSF of MS patients and controls. **a** Percentage of CSF samples with undetected miR-219 in cohort 1. **b** Scatter plot of miR-219 relative expression levels in CSF of individuals with detectable miR-219 levels of cohort 1. miR-219 levels were significantly decreased in SPMS and PPMS compared to controls. Levels were normalized using the geometric mean of miR-24 and miR-16. **c** Percentage of CSF samples with undetected miR-219 in cohort 2. **d** Scatter plot of relative expression levels of miR-219 in CSF of individuals with detectable miR-219 levels of cohort 2. Mean miR-219 levels in CSF of individuals with detectable miR-219 were similar in all groups. Levels were normalized using the geometric mean of miR-24 and miR-16. **e** Percentage of CSF samples with undetected miR-219 in cohort 3. **f** Scatter plot of miR-150 relative expression levels in CSF of individuals with detectable miR-150 levels of cohort 3. miR-150 levels were significantly increased in RRMS compared to PPMS, and in relapse-onset (RRMS, SPMS) compared to progressive-onset (PPMS). Levels were normalized using miR-204 levels. Black horizontal lines = median REL values ± interquartile range. * *p* < 0.05, ** *p* < 0.01. Abbreviations: RRMS relapsing remitting MS, SPMS secondary progressive MS, PPMS primary progressive MS, CIS clinical isolated syndrome, IIH idiopathic intracranial hypertension, AD Alzheimer’s disease, NHI neurologically healthy individuals, NIND non-inflammatory neurological diseases, IND inflammatory neurological diseases

We investigated the potential of miR-219 as a CSF biomarker discriminating MS from NHI in a first exploratory cohort. miR-24 and miR-16, surrogate markers for total miRNA expression, were detectable in all samples. In contrast, miR-219 could not be detected in all samples (Fig. 1a). Mean relative expression of miR-219 in individuals with detectable miR-219 was significantly lower in CSF of SPMS and PPMS patients compared to NHI (Fig. 1b). The odds ratio (OR) for a diagnosis of progressive MS (SPMS and PPMS) versus NHI was 20.8 (*p* = 0.0294) times higher if CSF miR-219 was undetectable (Table 2).

**Table 2:** Association between absence of miR-219 detection and MS

Subject comparisons	OR (95% CI)	*p* value two-tailed Fisher’s Exact test
Cohort 1		
MS (RRMS, PPMS, SPMS) vs. NHI	10.52 (0.5156–214.8)	0.0664
Progressive MS (PPMS, SPMS) vs. NHI	20.78 (0.9238–467.3)	*0.0294*
SPMS vs. NHI	23.80 (0.8934–634.0)	*0.0350*
Cohort 2		
MS (RRMS, PPMS, SPMS, CIS) vs. controls (NHI, AD, IIH, IND)	2.619 (1.173–5.847)	*0.0262*
Progressive MS (PPMS, SPMS) vs. controls (NHI, AD, IIH, IND)	3.636 (1.336–9.898)	*0.0165*
SPMS vs. controls (NHI, AD, IIH, IND)	4.667 (1.291–16.87)	*0.0314*
CIS vs. controls (NHI, AD, IIH, IND)	1.821 (0.4777–6.945)	0.4594
Cohort 3		
MS (RRMS, PPMS, SPMS) vs. controls (NIND, IND)	39.67 (4.104–383.4)	*0.0002*
RRMS vs. controls (NIND, IND)	26.25 (2.458–280.4)	*0.0025*
PPMS vs. controls (NIND, IND)	65.00 (2.237–1889)	*0.0047*
SPMS vs. controls (NIND, IND)	45.50 (3.479–595.1)	*0.0010*

Significant values are italicized

*OR* odds ratio, *CI* confidence interval, *CIS* clinically isolated syndrome, *RRMS* relapsing remitting MS, *SPMS* secondary progressive MS, *PPMS* primary progressive MS, *NHI* neurologically healthy individual, *AD* Alzheimer’s disease, *IIH* idiopathic intracranial hypertension, *NIND* non-inflammatory neurological disease, *IND* inflammatory neurological disease

We next sought to validate these findings in a larger, independent second cohort of MS patients, NHI and NIND, such as AD and IIH and IND. Again, miR-24 and miR-16 were detected in all samples, while the proportion of individuals with undetectable miR-219 was higher in the MS patients’ groups (Fig. 1c). However, mean relative expression of miR-219 in individuals with detectable miR-219 did not significantly differ in MS patients compared to controls in this cohort (Fig. 1d). There was no correlation between CSF miR-219 levels and age, disease duration, CSF erythrocyte count, or CSF leukocyte count. The OR for a diagnosis of MS (RRMS, SPMS, PPMS, CIS) versus controls was 2.6 (*p* = 0.0262) times higher if CSF miR-219 was undetectable, and the OR for a diagnosis of progressive MS versus non-MS was 3.6 (*p* = 0.0165) times higher if CSF miR-219 was undetectable (Table 2). The OR for a diagnosis of CIS versus controls (NHI, AD, IIH, IND) was not significant.

In a third cohort consisting of 90 MS patients and 22 controls, by omitting the pre-amplification step, we used a slightly different method to measure miRNA levels. Pre-amplification has a higher chance of resulting in false positive signals, especially in miRNAs with very low expression levels such as miR-219. The omission did result in less measurable total miRNA providing 38 MS and 8 controls with sufficient total miRNA to be included in the analysis of miR-219 expression. No differences in mean expression levels were observed between groups (data not shown), but again, individuals with undetectable miR-219 were more frequent in the MS patients’ groups (Fig. 1e). In this cohort, the OR for a diagnosis of MS (RRMS, SPMS, and PPMS) versus controls (IND and NIND) was 39.7 (*p* = 0.0002) times higher if CSF miR-219 was undetectable (Table 2). Significance was also observed when selecting specific MS groups (RRMS, SPMS, or PPMS) versus controls. miR-219 absence of detection was not correlated with gender or EDSS.

In addition to miR-219, we measured miR-150 levels in this cohort, as two groups independently showed discriminative power to differentiate MS patients from controls using this miRNA [29, 30]. This miRNA was thus used to validate the reliability of the miR-219 measurements in our study. miR-150 was detectable in 55 of the 112 CSF samples, while the frequency of undetectable miR-150 was similar between groups in terms of insufficient RNA yield, insufficient total miRNA level, or insufficient miR-150 level. Pair-wise comparisons showed significant differences in mean levels of miRNA-150 between RRMS and PPMS (*p* = 0.007), and between progressive-onset (PPMS) and relapse-onset (RRMS + SPMS) disease (*p* = 0.023), but there were no differences compared to controls (Fig. 1f). MiR-150 level was negatively correlated to age (*r* = − 0.298, *p* = 0.027). To estimate the biasing effect of age, analysis of variance (ANOVA) on the ranks was applied with age as covariate. This led to a loss of significance between RRMS and PPMS (*p* = 0.092), and between progressive-onset (PPMS) and relapse-onset (RRMS + SPMS) disease (*p* = 0.089). No significant differences were found between miR-150 levels in relapse-onset patients who did or did not use interferon-beta. miR-150 expression levels were not correlated with gender or EDSS.

## Discussion

Differentiation of neural precursor cells, including OPCs, is—amongst others—regulated by miR-219 [14–18]. This suggests that the differentiation failure of OPCs into mature oligodendrocytes in MS might be (partially) due to the lack of miR-219 expression. In this study, we investigated the levels of miR-219 in CSF in relation to MS diagnosis. MS patients show the highest rate of undetectable miR-219 compared to controls. In addition, there is a strong positive association between undetectable miR-219 and the diagnosis of MS (OR 2.9 and 39.7 in cohorts 2 and 3, respectively). We observed these findings in three independent cohorts, by applying two independent analytical procedures.

Our control groups consisted of neurologically healthy individuals and individuals with non-inflammatory neurological diseases (such as AD and IIH), but also consisted of individuals with inflammatory neurological diseases other than MS. This study therefore particularly looked into the specificity of miR-219 decrease in MS compared to a wide range of other neurological diseases for which a molecular biomarker does not necessarily have clinical relevance in differentially diagnosing MS. Future studies will therefore be necessary to further evaluate the performance of miR-219 in more clinically pertinent situations in which MS cannot be differentiated from other neurological diseases that mimic the MS phenotype.

In the third CSF cohort, we also measured miR-150 levels as an additional technical validation of the miRNA analysis. We found miR-150 to be significantly upregulated in relapse-onset (RRMS and SPMS) compared to PPMS patients. However, after correction for age, only trends remained for the differences in the mean miR-150 levels between groups. Previous studies on miR-150 in CSF by Bergman and coworkers and Quintana and coworkers [29, 30] did not discriminate between the different subtypes of MS, but found higher levels of miR-150 in MS patients compared to controls, which we could not confirm. Although the study by Bergman and coworkers also reported a significant correlation with age (*r* = −0.22, *p* = 0.002), they did not correct for this potential bias [29]. The study by Quintana and coworkers did not report any correlation analyses [30]. It is therefore unknown if the differences identified in either study would still be significant after correction for age. The effect of age on miRNA levels should require more attention in miRNA research, as age has been shown before to significantly modulate miRNA expression levels in CSF [31].

Our study further shows that detection of miRNAs in CSF is still challenging due to low levels of miRNAs in this body fluid leading to high amounts of samples with undetectable levels. The differences in the amount of undetectable samples from the different cohorts in our study can be explained by the different analytic methods used, which included a pre-amplification step in cohorts 1 and 2, not performed in cohort 3. This step was omitted in cohort 3 as pre-amplification has a higher chance of resulting in false positive signals, especially in miRNAs with very low expression levels such as miR-219. Detection might further be increased by increasing the total CSF volume used in the miRNA isolation procedures or by further optimization of the miRNA isolation procedure from biofluids to increase sensitivity.

miRNAs are interesting biomarker candidates as they are highly stable in body fluids [32] and, unlike proteins, the detection assays themselves are easily developed and multiplexed. Therefore, once the issues described above are evaluated further and more experience is gained, miRNAs, and more specifically, miR-219, may emerge as promising biomarkers for use in accurate diagnosis, prognosis, and treatment options in MS disease.

## Conclusion

We have been able to identify and validate absence of miR-219 detection in CSF of MS patients compared to controls, suggesting that it may emerge as a candidate biomarker for MS diagnosis.

## Abbreviations

AD: Alzheimer’s disease
ANOVA: Analysis of variance
CI: Confidence interval
CNS: Central nervous system
CSF: Cerebral spinal fluid
EDSS: Extended disability status scale
IIH: Intracranial hypertension
IND: Inflammatory neurological disease
miRNA: microRNA
MS: Multiple sclerosis
NHI: Neurologically healthy individual
NIND: Non-inflammatory neurological disease
OPC: Oligodendrocyte precursor cells
OR: Odds ratio
PP: Primary progressive
RR: Relapsing remitting
SP: Secondary progressive
